# Working Memory Capacity as a Predictor of Cognitive Training Efficacy in the Elderly Population

**DOI:** 10.3389/fnagi.2019.00126

**Published:** 2019-05-31

**Authors:** Olga Matysiak, Aleksandra Kroemeke, Aneta Brzezicka

**Affiliations:** ^1^Department of Psychology, SWPS University of Social Sciences and Humanities, Warsaw, Poland; ^2^Department of Neurosurgery, Cedars-Sinai Medical Center, Los Angeles, CA, United States

**Keywords:** working memory training, cognitive training, working memory capacity, older adults, dual N-back

## Abstract

Aging is associated with a decline in a wide range of cognitive functions and working memory (WM) deterioration is considered a main factor contributing to this. Therefore, any attempt to counteract WM decline seems to have a potential benefit for older adults. However, determination of whether such methods like WM trainings are effective is a subject of a serious debate in the literature. Despite a substantial number of training studies and several meta-analyses, there is no agreement on the matter of their effectiveness. The other important and still not fully explored issue is the impact of the preexisting level of intellectual functioning on the training’s outcome. In our study we investigated the impact of WM training on variety of cognitive tasks performance among older adults and the impact of the initial WM capacity (WMC) on the training efficiency. 85 healthy older adults (55–81 years of age; 55 female, 30 males) received 5 weeks of training on adaptive dual N-back task (experimental group) or memory quiz (active controls). Cognitive performance was assessed before and after intervention with measures of WM, memory updating, inhibition, attention shifting, short-term memory (STM) and reasoning. We found post-intervention group independent improvements across all cognitive tests except for inhibition and STM. With multi-level analysis individual learning curves were modeled, which enabled examining of the intra-individual change in training and inter-individual differences in intra-individual changes. We observed a systematic and positive, but relatively small, learning trend with time. Moderator analyses with demographic characteristics as moderators showed no additional effects on learning curves. Only initial WMC level was a significant moderator of training effectiveness. Older adults with initially lower WMC improved less and reached lower levels of performance, compared to the group with higher WMC. Overall, our findings are in accordance with the research suggesting that post-training gains are within reach of older adults. Our data provide evidence supporting the presence of transfer after N-back training in older adults. More importantly, our findings suggest that it is more important to take into account an initial WMC level, rather than demographic characteristics when evaluating WM training in older adults.

## Introduction

Advances in medicine and public health, in combination with rising standards of living, have lengthened the human lifespan. Increases in life expectancy, along with decreasing fertility rates, have led to the growth of a number of older adults in most populations. These days, a typical 60-year-old has many more years to live than a person of the same age 100 years ago (Office for National Statistics, 2017). This is unquestionably a positive change. However, there are also challenges associated with it. The most common effect concerns cognitive proficiency that is known to naturally decline with increasing age (Park and Payer, 2006; Salthouse, 2009; Harada et al., 2013). At the same time, new experiences and knowledge provide a potential for learning. This contrast between decline and learning is reflected in heterogeneous changes in cognitive performance that occur with age: while some of its aspects decline substantially, others may stay preserved. As previous empirical studies have indicated, age-related cognitive decline concerns especially fluid capacities (Salthouse, 2006; Hertzog et al., 2008; Lindenberger, 2014). Impairments are observable in measures of processing speed (Salthouse, 2009; Wahl et al., 2010), cognitive inhibition (e.g., Lustig et al., 2007; Rey-Mermet and Gade, 2018), processes of shifting (between tasks or mental sets) (meta-analysis: Wasylyshyn et al., 2011) and working memory (WM) (Kramer et al., 1999; Park et al., 2002; Klencklen et al., 2017). According to a definition by Miyake et al. (2000), above-mentioned functions constitute executive functions (EF) and are crucial for cognitively demanding day-to-day activities such as planning, reasoning, problem-solving, reading comprehension, and more general aspects of fluid intelligence (Cowan, 2010; Thompson et al., 2013). Therefore, all attempts to counteract the decline in EF are potentially beneficial for older adults and especially, cognitive training programs. WM seems to be a reasonable target for a cognitive training, given that it is perceived as a central component of general cognition and is inherently engaged in all higher-level cognitive activities. WM is referred to a moment-to-moment cognitive processing and temporary information storage (Logie et al., 2015). Studies have shown that the capacity of WM could be an indicator of performance in several other cognitive tasks: from simple attentional tasks (Kane et al., 2001; Bleckley et al., 2003; Fukuda and Vogel, 2009) to tasks requiring more compound capabilities, such as reading comprehension (Daneman and Carpenter, 1980), reasoning and problem solving (Kyllonen and Christal, 1990; Fry and Hale, 1996; Barrouillet and Lecas, 1999; Engle et al., 1999), as well as executive functioning in everyday life (Kane et al., 2007; Nagel and Lindenberger, 2015). Accordingly, one could expect that training-based increases in WM efficiency will be reflected as improvements in several other functions.

However, the effectiveness of WM training has been an ambiguous and passionately debated issue lately, especially the aspect of post-training growth in untrained abilities. A number of meta-analyses and reviews have addressed this matter (for meta-analysis see: Melby-Lervåg and Hulme, 2013; Karbach and Verhaeghen, 2014; Au et al., 2015; Schwaighofer et al., 2015; Melby-Lervåg et al., 2016; Soveri et al., 2017; for reviews see: Lövdén et al., 2010; Shipstead et al., 2010, 2012; Melby-Lervåg and Hulme, 2013; Shinaver et al., 2014; Au et al., 2016; Dougherty et al., 2016; Weicker et al., 2016). Initial enthusiasm about WM training effectiveness on fluid intelligence evoked by the results from Jaeggi et al. (2008); (see also: Lövdén et al., 2010), waned. In 2013 other researchers argued that “…there was no convincing evidence of the generalization of WM training to other skills” (Melby-Lervåg and Hulme, 2013, p. 270). Shinaver et al. (2014) responded with the meta-analysis showing highly significant effects of WM training on advancing verbal WM and visuospatial WM and thus confirming the existence of the near transfer. In 2015 another team (Au et al., 2015) reaffirmed those findings by showing a significant increase in fluid intelligence as a result of WM training. This conclusion was in line with results presented by others (Karbach and Verhaeghen, 2014; Schwaighofer et al., 2015). In 2016 Melby-Lervåg and Hulme, previously skeptical, presented a meta-analysis with positive post-training effects (Melby-Lervåg et al., 2016). Following training, there were reliable improvements in performance on verbal and non-verbal working-memory measures identical or similar to the trained tasks. However, in terms of the far transfer, there was no convincing evidence of improvements, especially when working-memory training was compared to an active-control condition. Also, Weicker et al. (2016) found small transfer effects to cognitive control and attention, but no transfer to long-term memory or reading comprehension. McCabe et al. (2016) went further and concluded their review with the idea that WM trainings, in the form that they are currently implemented, shall not be effective. However, the latest reports and meta-analysis are more hopeful. Soveri et al. (2017) observed medium-sized transfer effects to untrained N-back tasks and rather small effects for other WM tasks. However, overall pre-post training gains are medium sized and significantly higher than in control groups (Piryaei and Ashkzari, 2017).

Working memory training studies examined the role of age in explaining the benefits of training mostly by comparing young and older adults (see Borella and De Ribaupierre, 2014; for a review). Likewise, these results are ambiguous. Part of the studies reported larger enhancements in younger than in older adults (e.g., Dahlin et al., 2008; Schmiedek et al., 2010; Dorbath et al., 2011; Brehmer et al., 2012; Heinzel et al., 2014; Zinke et al., 2014). Other suggested that training gains are comparable (Li et al., 2008; Richmond et al., 2011; Bürki et al., 2014; von Bastian and Oberauer, 2014; Zajac-Lamparska and Trempała, 2016). As showed by Borella et al. (2017), if near or far transfer effects existed, the studies found either no differences between the two age groups, or larger effects in young adults than in older adults. Regarding the long-term effects, only two reviewed articles noted similar training improvements for both young as well as older participants, and only one found near and far transfer effects. However, larger long-term training-specific gains for young adults, and comparable long-term near transfer effects were also documented (Li et al., 2008). Among the studies focusing only on older adults, the ones that found significant training gains and transfer effects, were especially those involving young-old participants (<74 years old). Studies that included old-old participants (75–87 years old) or those that considered a broad age range reported mixed findings in terms of specific and transfer training gains. Likewise, according to the meta-analysis by Karbach and Verhaeghen (2014), cognitive interventions resulting in positive effects are those referring to near transfer. Far transfer observed in pre-to-post-training gains was significantly higher when improvements were compared to passive control group. Nonetheless, when analyzing group differences only at post-test level, profits were significantly bigger regardless of type of control group.

There have been mixed reports on the matter of the efficacy of WM training in aging, what makes it necessary to identify factors that are involved in modulating training benefits. We can specify the numerous elements that are believed to predict the benefits of memory training (e.g., Verhaeghen and Marcoen, 1996) and may also play a role as modulators of WM training outcomes (Bürki et al., 2014). The most prominent among them seems to be general cognitive ability and baseline cognitive resources. In an aforementioned work, Borella et al. (2017) showed that participants who had a higher initial cognitive performance and/or were younger were more likely to improve after the training. Interestingly, for more passive tasks (i.e., the Forward Digit Span – short term memory task) they observed a compensation effect: older subjects with lower initial vocabulary scores and weaker WM performance benefited more from the WM training. The same pattern was observed in the test similar to the trained task: participants with poorer achievements in a task based on crystallized intelligence – determined with the vocabulary test, benefited more than those with higher vocabulary scores. According to the authors, such pattern of results suggests that knowledge can counterbalance age-related decline (e.g., Baltes, 1987). These findings are considered an evidence for the compensation effect, which states that lower functioning individuals will have greater training benefits than high-functioning individuals, because they have higher learning potential (Olazarán et al., 2004). This was also confirmed in other studies (Ranganath et al., 2011; Zinke et al., 2012, 2014; Bürki et al., 2014). At the same time, the contrary hypothesis: so-called Matthew effect, exists. This phenomenon, popular in educational research (e.g., Bakermans-Kranenburg et al., 2005), refers to the observation that individuals with high initial ability are more likely to improve their skills to an even greater extent.

Taken together, there are solid foundations to believe that baseline cognitive resources could be crucial for WM training gains. It is, however, surprising how little reports have been published on this topic (e.g., Bürki et al., 2014; Foster et al., 2017). In fact, even large individual differences in initial cognitive resources, especially across older adults, are often neglected in the analyses of intervention research (Kliegel and Bürki, 2012). For this reason, this study investigated the influence of initial cognitive resources on WM training’s effectiveness in healthy older adults. We verified the possibility that demographic factors such as age, gender, education level or occupational activity of older adults could modify the training outcome. We compared a computerized, home-based dual N-back training program (experimental group) and an active control training based on a memory quiz (active control group). Including an active comparison group, instead of passive one, allowed for controlling every other element, except for the task used in the training (training content). The quiz task served as an active comparison since no impact of a semantic memory training (that refers to general knowledge of facts, ideas, and concepts) on WM has been found so far (see e.g., Li et al., 2016).

## Materials and Methods

### Participants

A total of 85 volunteers (55 female and 30 male) who matched inclusion criteria, aged 60 years and older (mostly from Warsaw, Poland) were recruited from Internet advertisements, Universities of the Third Age and social events for elderlies. Exclusion criteria were: history of brain trauma, use of medication known to affect cognition, progressive neurological diseases or severe hear or eyesight deficits. Participants were additionally screened for cognitive impairments [people with scores below 27 on the Mini-Mental State Examination (Folstein et al., 1975) were excluded from participation in the study]. 83 participants attended pre- and post-training tests. All seniors completed 25 training sessions although, as a result of technical problems, data from one participant was not recorded at all, same as information from 2 training sessions from another participant. For two seniors OSPAN task results from first measurement were improperly saved, therefore were excluded from analyses. Applicants were randomly divided into two training groups.

### Procedure

**FIGURE 1:**
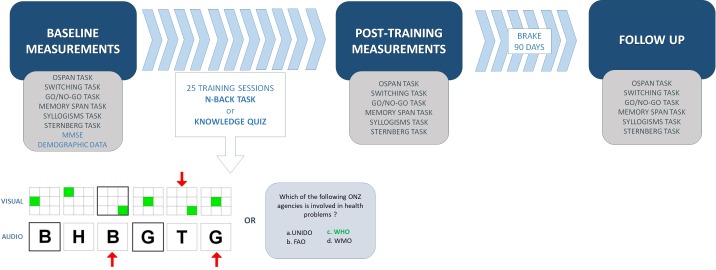
Study design with examples of a training tasks.

This study was carried out in accordance with the recommendations of the SWPS University of Social Sciences and Humanities Ethics Committee with written informed consent from all participants. All participants gave written informed consent in accordance with the Declaration of Helsinki. The research consisted of three steps (sessions): (1) Pre-training measurement of cognitive functions, (2) Training sessions, and (3) Post-training evaluation of cognitive performance (within a week following the last training) (Figure 1). Experimenters were present in all pre- and post-training meetings that took place in the laboratories of the SWPS University in Warsaw.

Working memory training was based on adaptive version of the dual N-back task. Active control group trained on semantic memory with the authorial Quiz Task. None of the training tasks were administered during evaluation. However, N-back tasks were discussed in detail with each participant, to ascertain that task objectives are comprehensible. Both training groups attended exactly 25 on-line training sessions spanning over 5 weeks. There were no restrictions concerning where or when practice should take place but the volunteers were obligated to accomplish the minimum of five training sessions per week and no more than one session per day. Subjects also needed to attend all of the sessions to be compensated and included in the analysis. The total compensation for each participant completing the experiment was 150 PLN (∼$40). Although participants were asked to not engage in other forms of “brain training” during the study period, we were unable to control for such potential contamination.

### Training Programs

#### Training Task 1 – Dual N-Back Task

An adaptive dual N-back task is a complex WM training program that simultaneously recruits auditory and visual attention, maintenance, and updating processes introduced by Jaeggi et al. (2008) One dual N-back session consisted of 15 rounds of 20 + N trials. Prior to the start of the actual task the participants were briefly instructed on the task at level *N* = 2. The visual stimuli were green squares presented in one of nine locations in a 3 × 3 matrix. Alphabet letters served as auditory stimuli. Each trial consisted of a 500-ms item presentation followed by a 2500-ms interval, during which the participants were supposed to respond. Volunteers were instructed to answer with a keyboard whenever the current stimuli matched the target stimuli presented N trials back. The current stimuli could match the target visual (response with left hand) or auditory stimulus (response with right hand) or both (response with both hands simultaneously). The N-back level was fixed at *N* = 2 for the first block, after which the N level for the current block was determined by the correctness of answers in the previous block. If accuracy fell below 85%, difficulty would not increase. Participants were informed of the N-back level before the start of each block. Reaction times (RTs) and accuracy (ACC) measures were obtained for each trial.

#### Training Task 2 – Memory Quiz

The active control group task comprised of 135 questions, which engage semantic memory. The questions were based on the material collected from the Internet. The training regime consisted of 25 sessions with 15 random questions each. The trainee was to answer the questions presented one after another. There was no time limit to read the question but after selecting the “answer” button the participant had to choose one of the four given possibilities within 40 s. The feedback about correctness was provided. Each session included 5 questions presented in the previous test and 10 new.

### Outcome Measures

Baseline and post-training neuropsychological assessment involved cognitive tests estimating WM, attention switching, processing speed and fluid intelligence. Measures were chosen on the basis of their use in previous investigations of WM training, their ability to reliably measure the cognitive domains of interest, and their sensitivity to age-related differences in cognitive performance. Each measure is described in detail below.

#### Operation SPAN Task

We used computerized version of original OSPAN Task (Turner and Engle, 1989). During each trial of this complex span task participants had to validate the results of simple equations and memorize a set of letters (F, H, J, K, L, N, P, Q, R, S, T, Y) at the same time. There were three practice sections: (1) Letter span: a group of letters appeared on the screen one by one, for 1000 ms each (in all experimental conditions). Participants were required to recall and choose the letters from the presented matrix, in the same order as they were presented. There was no time limit for this part. (2) Mathematical equation: Participants were instructed to calculate the results of the operations displayed on the screen (e.g., 3^∗^5 – 1 = ?) as quickly as possible and decide whether the presented on the next screen answer is correct (e.g., 16 – True/False?). During this section the program estimated individual time for participants required to solve the calculations. This time (plus 2.5 SD) was then used as the maximum time allowed for the mathematical portion in the main part of the task. (3) Mixed practice: Participants performed both subtasks, just as they would do in the real block of the attempts. Because of the dual-task character, complex span tasks put severe demands on EFs: apart from the updating of the WM content, subjects have to shift between the two subtasks and inhibit currently irrelevant information (Conway et al., 2002). A main task was identical to mixed practice section. This task is highly correlated with other measures of WM and fluid intelligence (Unsworth et al., 2005). Furthermore, it requires immediate attention, manipulation of information within immediate memory span and information updating. Therefore, OSPAN’s results may be reckoned as a complex measure of a cognitive reserve based on the WM capacity.

#### Syllogisms Task

Participants solved a computerized version of the linear-order reasoning paradigm – Syllogisms Task. This task consisted of set of premises that constituted logical chain of relations, and five questions about those relations. Three pairs of relations were studied in separate trials, each premise contained a pair of letters and information about a relationship between them e.g., “A > B,” “B > C,” and “C > D.” An integrated mental model representation (Johnson-Laird, 1983) of such a set of pairs would always be in the linear order “A > B > C > D.” There were two phases of the task – learning and testing. In the learning phase of the reasoning condition participants had to integrate presented information from three premises, where the first two were not related until the third premise appeared (A > B was followed by C > D and in the end by B > C). Immediately after presentation of the three pairs, we tested participants on all possible pairs within the order, i.e., AB, BC, CD (adjacent pairs, exactly the same as those that had been seen in the learning phase), AC, BD (two-step relations, not seen before and requiring integration of information), and AD (end point relation, not seen before and requiring integration of information), by prompting participants with statements in either a correct (e.g., “A > D”) or false format (e.g., “D > A”) and asking them for a speeded verification. We used capital letters as stimuli in all tasks instead of whole sentences in order to avoid linguistic connotations, and the symbol “>” was additionally presented to indicate the relation between elements. The arrangement of the letters was randomized in order to minimize possible interference induced by implied alphabetical ordering of letters. In the testing phase participants had to decide whether the questions about relations between letters were true (right button) or false (left button). In the easy condition premises were displayed one after the other (the subsequent premises had common element: e.g., B > V; V > M; M > A) in the difficult condition order of premises presentation was mixed (e.g., B > V; M > A; V > M). Questions about adjacent pairs probed memory and questions about two-step relations and end-point relations asked about information integration ability. The accuracy and response time (RT) for each question in both conditions were measured.

#### Memory SPAN Task

A digit-span task was used as a measure of WM storage capacity. During each trial of this task participants were presented with a series of letters, which elongated with each attempt. The stimuli were presented in the center of the screen for 1,500 ms, followed by a 500-ms interval. At the end of each trial, the participants were asked to recall between 3 and 6 last elements (maximum length of the series were 9). The participant’s span is the longest number of sequential digits that can accurately be remembered. Accuracy of recollection for each span was calculated.

#### Sternberg’s Task

Participants performed a computerized version of Sternberg’s paradigm (Sternberg, 1966). Each trial consisted of 2 to 5 white digits presented on a black screen in a sequence (1200 ms each). After maintenance period (2500 ms after the last digit) a yellow digit (target) appeared and participants had to indicate whether displayed sequence contained this digit or not (by pressing the adequate button). Task sessions were divided into equally distributed positive (“in” – probe present in the memory sequence) and negative (“out” – probe not present in the memory sequence) attempts. There were 120 experimental trials in total, preceded by 15 trials of the training. Accuracy of answers was measured.

#### Attention Switching Task

We reused procedure proposed by Dobrowolski et al. (2015), created on the basis of the tasks used by Colzato et al. (2013), with target stimuli adapted from Huizinga et al. (2006). Participants reacted as fast as possible to two kinds of requirements of the task. They had to decide if the figure is a square or a rectangle (condition 1 – global) or if the figure consists of squares or rectangles (condition 2 – local), depending on the presented cue. Global figures were comprised of smaller, local figures. Two training blocks were administered (each of 50 trials) with constant instruction across all trials. Answer was given by pressing “left button” for squares and “right button” for rectangles. Experimental block consisted of 160 trials in which participants switched between global and local. The measure of switch cost, based on differences in reaction times for conditions, was calculated.

#### Go/No-Go Task

The go/no-go is a cognitive task aimed at determining the ability of an individual to inhibit a response deemed inappropriate. Participants had to respond with a keyboard as fast as possible to the target stimulus (letter “X”) presented in the center of the screen, and to suppress the reaction for any other letter. The task consisted of two conditions based on ratio of go/no-go trials: easy- where ratio was 70/30, difficult – with ratio equal 50/50 (when half of the target letters came after target letter and the half came after any other letter). The trials were displayed on a white screen and composed of a 250 ms fixation point (white +), the stimulus presentation (a letter) for 1250 ms and a fixed inter-stimulus interval of 2000 ms. We considered the go/no-go task as a measure of cognitive inhibition efficiency.

### Statistical Analyses

All analyses were conducted using IBM SPSS Statistical Package for the Social Sciences (SPSS) version 24 and using Matlab custom scripts. For tasks that controlled the reaction time (Sternberg’s, Switching, Go/no go, OSPAN tasks) outlying trials (exceeding cut-off of 2.5 standard deviations, individually calculated for each participant) were removed. A significance level of *p* < 0.05 was adopted for all conducted tests and *post hoc* comparisons. All *post hoc* between-groups comparisons were Bonferroni corrected. Repeated measures multivariate analysis of variance (RM-MANOVA) was used to test for differences in training groups (N-back training, Quiz training) over time (pre-, post-training) across the dependent variables (outcome measures).

In order to find a more refined method to observe learning process, i.e., the unique effects of performance in the 1st training session and cumulative change in training improvements over time, we decided to conduct multilevel modeling (MLM). MLM provides the best parameter estimates while accommodating the hierarchical structure of the data: repeated measurements (level 1) nested within participants (level 2) (Bolger and Laurenceau, 2013). The MLM analysis dataset consisted of 42 (participants) × 25 (sessions) = 1,050 observations. Both fixed (the regression intercept and slope for the average person) and random effects (between-subject variability around the average) were examined. In Model 1, the change in N-back task scores over time was modeled represented by the number of a training session as a predictor (time). To test predicting and moderating effects of demographics (age, sex, education level, occupational activity) as well as the influence of a baseline OSPAN score (between-person predictors – level 2) on within-subject variation (level 1) in N-back training additional models (2 to 6) were calculated. To avoid multicollinearity, all predictors were tested separately. Initial OSPAN score was categorized and two groups were created: (1) group with higher than mean scores (high initial OSPAN), (2) group with average and lower than average scores (low initial OSPAN). Age was grand-mean centered and dummy-codes were created for categorical demographic variables. In all models, a quadratic effect for the slope (besides linear one) was tested and subsequently removed because its fixed effects and variance components were not significant. The time variable was centered at 1st day of the training. The restricted maximum likelihood (REML) was used as the estimator. Goodness of fit for the models was based on –2 Restricted log likelihood ratio (-2LL) and the Akaike Information Criterion (AIC). The first-order autoregressive [AR(1)] covariance structure was used for the models, given the common proximal autocorrelation in the daily data (Kwok et al., 2008).

## Results

### Participants Characteristics

There were no group differences in the demographic data, but we found variance between groups in the baseline measure of general cognitive functioning – OSPAN task. Table 1 presents participants’ characteristics.

**Table 1:** Participants characteristics at baseline.

	N-back group	Quiz group	Statistics	
			Test	*p*-Value
*N*	42	42	X^2^(1) = 0.12	0.914
Age	*M* = 65.9	*M* = 67.5	*t*(82) = 2.342	0.199
Sex (female/male)	28/15	27/15	X^2^(1) = 0.006	0.936
Education (higher/secondary)	27/16	21/21	X^2^(1) = 1.414	0.234
Occupational activity (active/retired)	13/29	10/32	X^2^(1) = 539	0.463
OSPAN scores (high/low)	26/17	11/31	X^2^(1) = 10.15^∗^	0.001
OSPAN scores [absolute score]	*M* = 15.31	*M* = 9.07	*t*(80) = 0.322	0.233


*Group (N-back – training group; Quiz – active control group) differences at baseline. Parenthesis () for categorial variables, [] for continuous data. ^∗^Statistical significance: *p* < 0.05.*

### Training Tasks Results

Training results were analyzed in the N-back group only as the quiz group did not undergo a cognitive training and executed tasks of equal difficulty through the whole length of the study. Training progress in the WM training group was based on maximum N-back level achieved in each of 25 training sessions. All participants started at level *N* = 2 and could increase this number by good execution (80% of accuracy in the block) of the task or have it set to the *N* = 1 level because of poor performance in task (below 65% of accuracy in the task). Throughout training, 21.4% of the volunteers never surpassed entering level, other 28.6% reached maximum 3-back, and almost half of the group managed to attempt 4-back (47.6%). Only one participant reached 5-back. The mean maximal N-back level achieved through the whole training was 3.31 (*SD* = 0.14), and on the last day of training – 2.9 (*SD* = 0.15). The difference in average N-back level achieved from the first to last day of training was statistically significant, *t*(40) = 5.89, *p* < 0.001.

**FIGURE 2:**
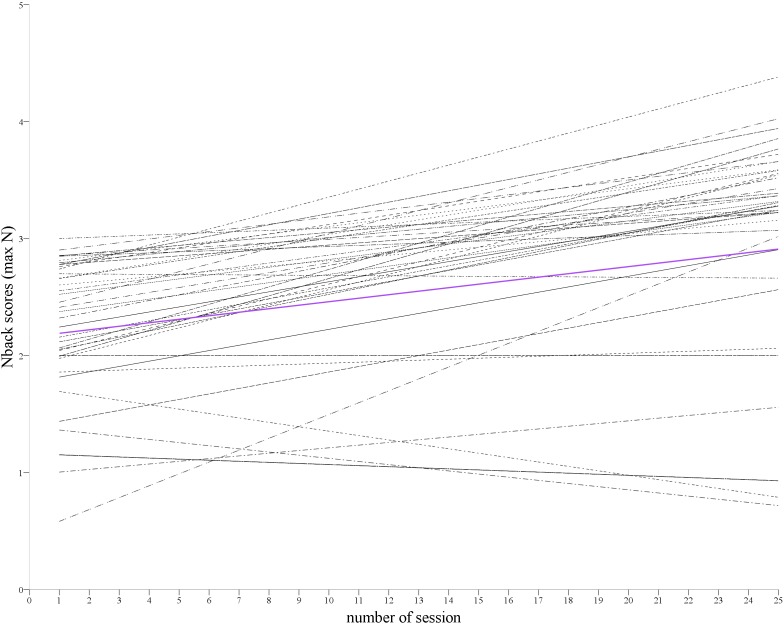
N-back training effectiveness. Change in training task scores (N-back) over time (training sessions) estimated for each participant (black lines).

Results of MLM indicated that the initial N-back level was 2.16 on a 1+∞ scale and showed a 0.03 unit increase over 25 days of training (see MODEL 1, Figure 2 and Table 2). In addition, all random effects were significant, pointing to the between-person variability in intercept, slope, and positive correlation between max n-level achieved in the first time of a training and upswing in the task results over time.

**Table 2:** Multilevel anlaysis of the training data (n-back task).

	MODEL 1		MODEL 2		MODEL 3		MODEL 4		MODEL 5		MODEL 6	
	+ change in time in Nback task		+ age as a predictor		+ sex as a predictor		+ education level score as a predictor		+ occupational activity as a predictor		+ Initial OSPAN	
						
	*b*	(s.e.)	*b*	(s.e.)	*b*	(s.e.)	*b*	(s.e.)	*b*	(s.e.)	b	(s.e.)
**Fixed effects**												
Intercept	2.165	(0.088)***	2.162	(0.093)***	2.216	(0.151)***	2.238	(0.111)***	2.278	(0.158)***	1.928	(0.143)***
Time – linear (centered at 1st day)	0.031	(0.005)***	0.028	(0.005)***	0.036	(0.008)***	0.035	(0.006)***	0.037	(0.008)***	0.016	(0.007)*
Ageˆ	—	—	0.010	(0.018) n.s.	—	—	—	—	—	—	—	—
Sex∼	—	—	—	—	–0.008	(0.188) n.s.	—	—	—	—	—	—
Education level∼	—	—	—	—	—	—	–0.204	(0.186) n.s.	—	—	—	—
Occupational activity∼	—	—	—	—	—	—	—	—	–0.131	(0.191) n.s.	—	—
Initial OSPAN score ∼ (high/low)	—	—	—	—	—	—	—	—	—	—	0.038	(0.183)*
Time × age	—	—	–0.002	(0.001)°	—	—	—	—	—	—	—	—
Time × sex	—	—	—	—	–0.008	(0.009) n.s.	—	—	—	—	—	—
Time × education level	—	—	—	—	—	—	–0.011	(0.009) n.s.	—	—	—	—
Time × occupational activity	—	—	—	—	—	—	—	—	–0.008	(0.010) n.s.	—	—
Time × initial OSPAN score	—	—	—	—	—	—	—	—	—	—	0.026	(0.008)**
**Random effects ([co-]variances)**												
Level 2 (between-person)												
Intercept	0.285	(0.073)***	0.301	(0.079)***	0.300	(0.006)***	0.292	(0.076)***	0.283	(0.075)***	0.286	(0.075)***
Time – linear	0.001	(0.001)***	0.001	(0.001)***	0.001	(0.001)***	0.001	(0.001)***	0.001	(0.001)***	0.001	(0.001)***
Intercept and time	0.006	(0.003)*	0.006	(0.003)*	0.006	(0.003)*	0.006	(0.003)*	0.005	(0.003)°	0.004	(0.002)°
Level 1 (within-person)												
Residual	0.151	(0.009)***	0.149	(0.009)***	0.150	(0.009)***	0.151	(0.009)***	0.153	(0.009)***	0.149	(0.009)***
Autocorrelation	0.339	(0.039)***	0.336	(0.040)***	0.338	(0.039)***	0.338	(0.039)***	0.336	(0.039)***	0.328	(0.040)***
**Model fit**												
–2 log likelihood *(y2)*	1069.32		1063.69		1089.51		1088.24		1077.91		1046.37	
Akaike’s Information Criterion (AIC)	1083.32		1073.69		1099.51		1098.24		1087.91		1056.37	


Results from multilevel modeling. Unstandardized regression coefficients listed with standard errors in parenthesis, °*p* = 0.1, *p < 0.05, **p < 0.01, and ***p < 0.001. All *p*-Values are two-tailed except in the case of variances, where one-tiled *p*-Values are used (because variances are constrained to be non-negative). ^∧^The predictors are mean centered. ^∼^The predictors are dichotomous.

### Predictors of a Training Effectiveness

**FIGURE 3:**
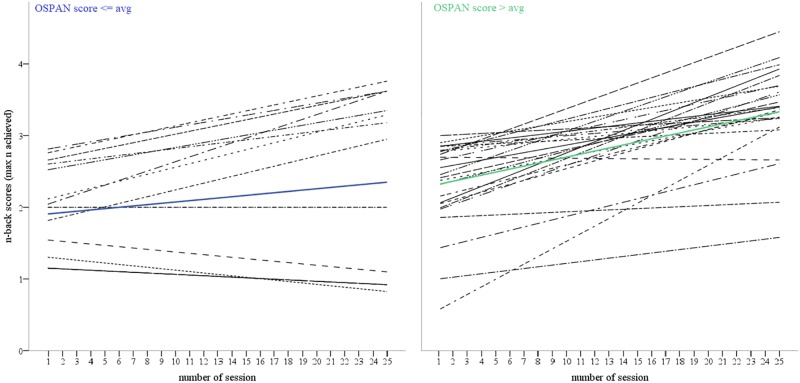
Change in training task scores (N-back) over time (training sessions) presented for high and low performers in baseline OSPAN task measurement.

The results of MLM for models 2–6 (Table 2) revealed that none of demographic variables was predictive neither of the initial level of max N-back nor of its change over time. Only baseline OSPAN performance turned out to be a significant predictor of the N-back result at the first day of training and moderator of the whole training course. Both groups, with high versus low OSPAN scores, showed an initial N-level of approximately 2.00 units on a 1+∞ scale (low OSPAN = 1.93; high OSPAN = 1.93 + 0.38 = 2.31). After training, the participants with low initial OSPAN scores showed a 0.01 unit increase in N-back level, whereas the participants with the high initial OSPAN scores showed a 0.01 + 0.03 = 0.04 unit increase in N-back training. The initial performance of the OSPAN task clearly affects the trajectory of improvement during the training: people with a higher initial OSPAN score have significantly higher N-level achievement in the 1st session and a steeper learning curve in training (*p* < 0.001), which is presented in Figure 3.

## Outcome Measures Results

To determine whether baseline cognitive performance was comparable across training groups, we conducted a MANOVA with all the pretest measures as the dependent variables and the training group as the between-subjects factor. The main effect of the group was not significant, *F*(8,71) = 1.815, *p* = 0.88, $\eta_{p}^{2}$ = 0.17. In addition, almost all of the Bonferroni corrected *post hoc* between-groups comparisons for outcome measures were non-significant. We noted pre-training group discrepancy in one task. The N-back group showed better baseline performance in the OSPAN task than the active control group (Mdiff = 6.79, *p* = 0.007, $\eta_{p}^{2}$ = 0.09).

Transfer effects were analyzed for each outcome results for both training groups over time (pre-, post-training) with RM-MANOVA using tasks’ performance as dependent variables, and training group and measurement points (pre- versus post-training) as independent variables. Table 3 presents results of this analysis.

**Table 3:** Statistical evaluation of the change in outcome measures.

	Pre- to post-training effect			Training group effect			Interraction effect (time × training group)		
	Mdiff^1^	*F*(1,83)	$\eta_{p}^{2}$	Mdiff^2^	*F*(1,83)	$\eta_{p}^{2}$	Mdiff^3^	*F*(1,83)	$\eta_{p}^{2}$
OSPAN task	3.05°	3.67	0.04	8.47*	13.01*	0.14	Nback: 5.00* Quiz: 1.10	1.49	0.19
Syllogisms task	0.10*	31.22	0.27	–0.01	0.01	<0.001	1:0.008 2: –0.013	0.35	0.01
Memory SPAN task	0.03°	3.13	0.04	0.09*	7.72	0.09	1:0.09* 2:0.10*	0.04	<0.001
Sternberg’s task	0.02°	3.56	0.04	0.02	0.78	0.01	Nback:0.01 Quiz:0.03°	0.62	0.01
Attention switching task	–0.07*	5.79	0.07	–0.04	0.75	0.01	Nback: –0.08° Quiz: –.07	0.02	<0.001
Go/no-go task	0.01	0.01	<0.001	–0.01	0.21	0.01	1:0.01 2: –0.02°	2.82	0.03


^1^Mean difference (M session 2 – M session 1); ^*2*^Mean difference: M Nback – M Quiz; ^*3*^Mean difference for: (a) 1 session: M Nback – M Quiz, 2 session: M Nback – M Quiz (b) Nback: M session 2 – M session 1, Quiz: M session 2 – M session 1. *Statistically significant effect; °tendency toward significance

**FIGURE 4:**
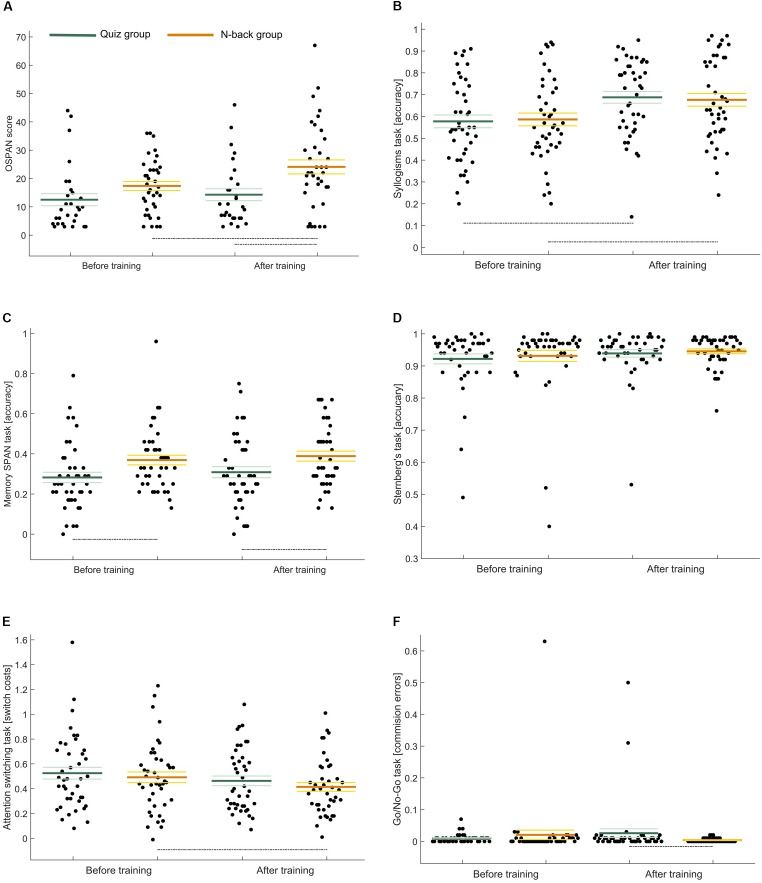
Pre-to-post training changes in measurements of: **(A)** working capacity – OSPAN task, **(B)** reasoning – Syllogisms task, **(C)** working memory span – running memory SPAN, **(D)** short-term memory – Sterberg’s task, **(E)** attention switching – switching task, **(F)** – cognitive inhibition – go/no-go task.

Repeated measures multivariate analysis of variance revealed post-training improvement in OSPAN performance (a strong tendency toward statistical significance: *p* = 0.06). However the initial disproportion between groups (higher results in the N-back group) remained. Although interaction effect *per se* was not statistically significant, *post hoc* comparisons revealed that only participants in the N-back group increased their OSPAN scores (*p* = 0.02) (Figure 4A). We have also observed a statistically significant post-training growth in syllogisms task results (*p* < 0.001), regardless of the type of intervention (Figure 4B). In the memory SPAN task the baseline assessments were slightly worse (*p* = 0.08) than after the intervention for all the participants. However, the analysis showed relatively large disproportion in results between groups: participants in the N-back training group achieved higher scores at baseline, as well as after training (Figure 4C). As could be predicted based on the RM-MANOVA results the post-training scores were also better in both groups in Sternberg’s task (a strong tendency toward statistical significance: *p* = 0.06). *Post hoc* comparisons revealed that the difference in task accuracy was better after training only in the quiz group (*p* = 0.06) (Figure 4D). Likewise, performance in attention switching increased with time (Figure 4E): switch cost was smaller after the training, which is reflected in the negative difference in means (*p* = 0.02). Pre- and post- training performance in the go/no-go task was dependent on the training group affiliation. Although none of the between-subject effects was statistically significant, we found a close to significance interaction (*p* = 0.09) (Figure 4F). Training groups did not differ in the performance on the first measurement, but after the training the participants exercising with the N-back task improved their results (fewer commission mistakes), while the quiz group recorded worse results after the training.

That means that performance in all outcome measures was improved by the intervention. However, only changes in the cognitive inhibition efficiency and WMC appeared to be positive and more pronounced after WM training.

## Discussion

The aim of the study presented here was to investigate the effects of WM training with the dual N-back task in healthy older adults from Poland, developing country with an aging population demography. The first analysis was focused on answering the question of whether there was a progress in the active training task itself. The results showed that most of the elderly participants were able to improve on the N-back task, although we identified a substantial number (21.4%) of them not being able to surpass the entering level of N during the whole training. The specificity of a dual N-back task makes it very challenging, particularly for older adults. Dual N-back simultaneously recruits auditory and visual attention, maintenance, and updating processes. Those are the exact cognitive functions that most commonly decrease with age (Wasylyshyn et al., 2011; Overdorp et al., 2016). At the same time not every senior experience cognitive decline at the same degree (Stern, 2009). There is a possibility that effect observed in physical training of older adults (Godde and Voelcker-Rehage, 2017) is virtual also for cognitive intervention and those, who had started with lower initial level of function trained, finished with a better post-training results. The multilevel analysis revealed a significant learning effect for the whole group that trained with the N-back task, meaning that, in general, our participants improved throughout the training course. As expected, there was an appreciable variability in the N-back task performance from the early beginning of the training to its end. Individuals with higher maximal N achieved at the first session improved faster than the others on consecutive training sessions (the effect with a high tendency to statistical significance). That indicate that the initial gap in performance between the participants increased with time. A similar result was reported by Foster et al. (2017). They exposed the relation between baseline WM agility and performance on the memory span training tasks, which suggests that a person’s ability to gain from WM training depends on this person’s prior level of intellectual skills. We explored this matter more closely via another multilevel analysis accommodating initial cognitive discrepancies and demographic variables as predictors of the improvement gained during the training process. The results showed that the preliminary score in OSPAN task, interpreted as a level of cognitive functioning based on the WM capacity assessment (WMC) was a strong predictor of change in the learning curve during the training. Participants characterized by higher initial WMC performed better in training from the very first day and had stepper learning curve in comparison to seniors with WMC below the average of the sample. Those observations are in line with our predictions and studies reporting Matthew effect in WM training interventions, where participants with initial higher ability benefited more from training on this ability as well as on new skills (e.g., Bissig and Lustig, 2007; Borella et al., 2010; Bürki et al., 2014; López-Higes et al., 2018; Kraemer et al., in press).

However, when compared to results supporting contrary hypothesis of compensation effect in cognitive interventions (Mondini et al., 2016; López-Higes et al., 2018) alternative interpretation of our results might be raised. Effects observed in our study could be explained by the mismatch between capacity for plasticity (“supply”) and “demands” of the environment, in accordance to the Lövdén et al. (2010) hypothesis. In our study the difficulty of the training task is a demand and one’s cognitive abilities are supplies. The discord appears when the demands exceed challenges that the cognitive system usually faces. In those situations individuals might just give up on the task, what could be noticed as a flat learning curve in N-back task. They could also develop task-specific strategies enabling to hurdle task requirements, nonetheless impossible to transfer to performance in post-training measurements. Such strategy should be reflected in worse post-training gains among those subjects. Unfortunately, the size of the sample in our study precludes any reasonable analysis on this matter. Some of the other studies, presenting results contrary to ours, verified post-training gains using the measure, which was one of our exclusion criterion – MMSE score (López-Higes et al., 2018). It is hence impossible for us to arbitrate how WM training improved the results of participants with low MMSE scores, since they were excluded from the study.

It is important to note that WMC was the only significant predictor of the training progress. None of the demographic variables was a significant predictor of the training effectiveness. This result contrasts with other studies showing that educational achievements (proxy of cognitive reserve) modulate training gains, and participants with lower educational level usually achieve better outcomes (Clark et al., 2016). However, this effect could be also explained by subjects’ age. Zinke et al. (2014) examined both initial WMC and age in the context of a WM task performance. They found that both factors – the initial WMC and age – contributed to the amount of a training gain, but age was a weaker predictor than the initial WMC. Thus, age influence on training outcomes probably reflects the sole effect of pre-training cognitive ability rather than a general cognitive decline over the life-span (von Bastian and Oberauer, 2014). It may be that including old-old adults would affect the magnitude of the effect of the age on training gains, because they experience a more noticeable cognitive decline than the young-old (Borella et al., 2007), regrettably our oldest participant was 81 years old. Nevertheless, these observations give foundations for further exploration of an influence of initial variability in cognition on training gains.

Our third analysis concerned the effectiveness of training through its impact on the measures of cognitive functioning. We expected post-training improvement in the tasks involving the same function as the one being trained (Operation SPAN Task, Sternberg’s Task, memory SPAN task) and insignificant training effects for tasks distantly related to WM (attention switching, syllogisms, and go/no-go tasks). According to Barnett and Ceci’s taxonomy (Barnett and Ceci, 2002) near transfer appears when training task and outcome measures are structurally similar. Executing N-back is likely to lead to improvements on similar in design and structure N-back task, as was demonstrated by Lilienthal et al. (2013) and von Bastian and Eschen (2016). With dual N-back task we were aiming to train a processes of WM, and verify if the process was improved afterward. That is the reason why we used the memory SPAN and OSPAN tasks, which are as far as possible from training task in design, however, they are still measures of the same cognitive construct. Therefore, improvement in those tasks should be seen more as indicator of the mid-transfer rather than near transfer. The third indicator of memory abilities – Sternberg task – is a measure of short-term memory (STM) capacity. That is why training influence on its scores is treated as an evidence of the far transfer. Likewise, a syllogistic reasoning task. As we predicted, analyses revealed significant post-training gains in memory SPAN task accuracy and operation SPAN scores only in an N-back group (near transfer). An opposite effect was found for Sternberg task – only the quiz group showed an improvement after training. With respect to the STM, it should be noted that the N-back task requires temporary storage, and training might increase short-term storage capacity in either the verbal or the spatial domain. Apparently, the discrepancy between verbal/spatial (N-back) and visual (Sternberg’s task) stimuli in the tasks obstructed post-training gains or the N-back tasks simply do not promote STM capacity. Such explanation could be supported by the lack of transfer to STM capacity, which was also found in several other studies (e.g., Owen et al., 2010; Lilienthal et al., 2013). To our surprise, the quiz training group showed improvement in this task. Although we did not exclude the possibility of observing positive changes after the semantic memory training, we assumed that they would be rather subtle. However, Kuo et al. (2018) demonstrated that significant post-training improvement in EF (such as attentional control engaged in simultaneous execution of WM task) could be observed after semantic memory training.

Further analysis revealed the lack of training effects on inhibitory control tasks in both training groups. The higher post-training results were found in the active control group, but pre-to-post training changes were insignificant in both groups. While interpreting this results, especially when the screened group consists of older adults, it is important to be aware of the conflict at the perceptual and/or motor level. Aging processes may differently affect motor and perceptual inhibition as well as the interplay between them. It is almost impossible to determine which of those two played a greater role in making the errors in go/no-go task when only behavioral measures were taken into account. Unfortunately, we did not control for our participants’ motor functions. It is also suggested that the increased noise in perceptual processing is a key factor for age-related memory reductions (Schneider and Pichora-Fuller, 2000). The decrease in sensory functioning may demand a greater cognitive effort to uphold sensory functioning, thus leaving less available resources for cognitive operations. It has been shown that older adults under quiet conditions perform at the same level as if younger adults were presented with information in auditory noise (Murphy et al., 2000). The quiz group, that trained with less cognitively demanding, semantic memory task at home (in a noise-uncontrolled environment) could be less challenged to overcome the influence of surrounding distractors. Therefore, it is possible that they developed different, and possibly less effective strategies eliminating signal-to-noise ratio influence on task performance than N-back group which was exposed to a severe attention load. Related to the inhibitory deficit theory are claims that the amount of attentional resources is the limiting factor in aging. Decades ago studies showed that, under divided attention conditions, younger adults lower their performance to similar levels as older adults under single-task conditions (Anderson et al., 1998). To proceed under these circumstances people need to allocate all attentional resources, and if they are not sufficiently available, the encoding and subsequent recall will be affected negatively. Regarding this, it is unsurprising that a positive post-training effect in attention switching task was found only in N-back group.

We would like to put the emphasis on the recent reinterpretation of WM interventions reporting positive effects of WM training relative to control groups. It revealed that observed transfer effects were present due to decreases in control group performance rather than reliable increases in training groups (Redick, 2015). That is why it is important to stress that our study showed lack of post-training reduction in outcome measurements in the quiz group, except for the go/no-go task. It is also worth noticing that the social engagement in general is considered to be the main factor in maintaining good intellectual health. We have to remember that all our participants (regardless of group belongingness) volunteered to the study. Correlational studies have shown that voluntary work protects against cognitive aging (reviews: Anderson et al., 2014; Guiney and Machado, 2018; Proulx et al., 2018), so we cannot exclude the possibility that any new and voluntary engagement might be intellectually beneficial for elderly people. Overall, our findings do fit well with the research suggesting that post-training gains are within reach of older adults. However, it is always advisable to achieve the best possible cognitive agility as early as possible and before aging deficits appear.

## Ethics Statement

This study was carried out in accordance with the recommendations of the SWPS University of Social Sciences and Humanities Ethics Committee with written informed consent from all participants. All participants gave written informed consent in accordance with the Declaration of Helsinki. The protocol was approved by the University Ethics Committee.

## Author Contributions

AB developed the theoretical framework and supervised the project, and corrected the manuscript. OM carried out the experiments and wrote the manuscript. OM and AK performed the analytic calculations. AK corrected the manuscript.

## Conflict of Interest Statement

The authors declare that the research was conducted in the absence of any commercial or financial relationships that could be construed as a potential conflict of interest.
